# Transmission of Foot-and-Mouth Disease Virus during the Incubation Period in Pigs

**DOI:** 10.3389/fvets.2016.00105

**Published:** 2016-11-21

**Authors:** Carolina Stenfeldt, Juan M. Pacheco, Barbara P. Brito, Karla I. Moreno-Torres, Matt A. Branan, Amy H. Delgado, Luis L. Rodriguez, Jonathan Arzt

**Affiliations:** ^1^Foreign Animal Disease Research Unit, Plum Island Animal Disease Center, Agricultural Research Service, United States Department of Agriculture, Greenport, NY, USA; ^2^PIADC Research Participation Program, Oak Ridge Institute for Science and Education, Oak Ridge, TN, USA; ^3^Monitoring and Modeling, Animal and Plant Health Inspection Service, Center for Epidemiology and Animal Health, United States Department of Agriculture, Fort Collins, CO, USA

**Keywords:** foot-and-mouth disease, foot-and-mouth disease virus, virus diseases, pigs, transmission, incubation, subclinical, preclinical

## Abstract

Understanding the quantitative characteristics of a pathogen’s capability to transmit during distinct phases of infection is important to enable accurate predictions of the spread and impact of a disease outbreak. In the current investigation, the potential for transmission of foot-and-mouth disease virus (FMDV) during the incubation (preclinical) period of infection was investigated in seven groups of pigs that were sequentially exposed to a group of donor pigs that were infected by simulated-natural inoculation. Contact-exposed pigs were comingled with infected donors through successive 8-h time slots spanning from 8 to 64 h post-inoculation (hpi) of the donor pigs. The transition from latent to infectious periods in the donor pigs was clearly defined by successful transmission of foot-and-mouth disease (FMD) to all contact pigs that were exposed to the donors from 24 hpi and later. This onset of infectiousness occurred concurrent with detection of viremia, but approximately 24 h prior to the first appearance of clinical signs of FMD in the donors. Thus, the latent period of infection ended approximately 24 h before the end of the incubation period. There were significant differences between contact-exposed groups in the time elapsed from virus exposure to the first detection of FMDV shedding, viremia, and clinical lesions. Specifically, the onset and progression of clinical FMD were more rapid in pigs that had been exposed to the donor pigs during more advanced phases of disease, suggesting that these animals had received a higher effective challenge dose. These results demonstrate transmission and dissemination of FMD within groups of pigs during the incubation period of infection. Furthermore, these findings suggest that under current conditions, shedding of FMDV in oropharyngeal fluids is a more precise proxy for FMDV infectiousness than clinical signs of infection. These findings may impact modeling of the propagation of FMD outbreaks that initiate in pig holdings and should be considered when designing FMD control strategies.

## Introduction

Foot-and-mouth disease (FMD) is a highly contagious and economically devastating disease that affects cloven-hoofed animal species. The infectious agent, foot-and-mouth disease virus (FMDV; genus *Apththovirus*, family *Picornaviridae*), is infectious at low doses and capable of rapid dissemination within susceptible animal populations (1). Clinical FMD is characterized by fever, lameness, and ptyalism concurrent with the occurrence of vesicular lesions in the oral cavity and on the feet. However, the clinical manifestations of FMD may vary greatly depending both on biological properties of the different virus strains and on the host species affected (2–4).

Although FMD-associated mortality rates among adult animals are generally low, the intensive countermeasures enacted to combat disease outbreaks in FMD-free countries often result in depopulation and destruction of large numbers of infected and susceptible animals (5–7). Large regions of the world, including Europe, Australia, North America, and parts of South America, are kept free of FMD by means of strict regulations on import of animals and agricultural products. Animal populations within these regions where prophylactic vaccination is not practiced are highly vulnerable to potential FMDV incursions due to the lack of herd immunity. As access to international trade markets for agricultural products is largely dictated by a country’s official FMD status, introduction of the disease into these regions will have massive financial and logistical implications for the agricultural sectors (8). Additionally, there are substantial ethical and environmental concerns associated with depopulation of large numbers of animals for the purpose of controlling potential FMD outbreaks.

The ability to efficiently combat FMD outbreaks in regions with highly susceptible animal populations is dependent on early detection of the incursion as well as the ability to efficiently trace and identify animals, herds, and premises that may have been exposed to the source of infection (9–12). The time elapsed from the first infection until the first case has been detected is generally referred to as the “high risk period” (13). As was seen during the extensive FMD outbreak in the UK in 2001, inability to detect infection during this early phase of an outbreak can result in substantial dissemination before appropriate countermeasures, such as animal movement restrictions, are enforced (14, 15). Two critical factors that complicate control of the early phase of an outbreak are potentially subtle or unapparent clinical signs of infection as well as disease transmission occurring during the incubation period, prior to the development of detectable clinical signs of FMD.

In a study by Charleston et al. (16), it was concluded that cattle exposed to FMDV by direct contact to infected cattle, were not infectious until, on average, 0.5 days after the appearance of clinical signs of FMD. Thus, the conclusion of this study was that transmission of FMDV during the incubation phase would not be likely to have a significant influence on disease dissemination in an outbreak situation. In contrast to this, a previous publication by Orsel et al. (17) concluded that substantial FMDV transmission may occur prior to onset of clinical signs in groups of cattle or pigs that are housed together. However, the latter publication also reported substantial differences in the occurrence of preclinical FMDV transmission depending on host species (cattle, pigs, or sheep) as well as on the age of the animals. While the occurrence of preclinical transmission was low within groups of young calves and lambs, it was substantially higher within groups of multiparous dairy cows as well as among 10–12 weeks old pigs (17).

Orsel’s study design allowed for an approximation of the extent of preclinical transmission that had occurred. However, it was not possible to determine at which specific times transmission had occurred or to estimate the onset of infectiousness in the donor animals. These limitations resulted from the utilization of data from previous experiments that were originally designed for other purposes (18–21). The ratios of preclinical transmission were estimated by determining the number of new infections [defined by detection of FMDV shedding in oropharyngeal fluid (OPF)] that occurred before the donors developed clinical signs of FMD.

Multiple investigations have demonstrated rapid and efficient transmission of FMDV within groups of pigs that are housed together during the clinical phase of infection (18, 22–29). However, to the best of our knowledge, there are no published works characterizing the onset or continuous progression of infectiousness in FMDV-infected pigs. The current investigation was designed to determine the onset of infectiousness in relation to the development of clinical disease in pigs infected with FMDV. This work provides a novel and detailed characterization of the time-dependent progression of FMDV transmission dynamics within groups of pigs. The demonstration of substantial preclinical transmission of FMDV may influence modeling of FMDV outbreak scenarios involving this host species and is highly relevant to the development of outbreak response strategies.

## Materials and Methods

### Virus

The virus used for this study was a cattle-derived strain of FMDV A24 Cruzeiro that had been passed once in pigs. Details of the generation and titration of the virus stock has been published previously (23).

### Animal Experiments

All animal studies were performed within the BSL-3Ag containment facility at the Plum Island Animal Disease Center. Experimental protocols were approved by the facility’s Institutional Animal Care and Use Committee, which functions to ensure ethical and humane treatment of experimental animals. All animals were castrated male Yorkshire pigs, weighing approximately 30 kg upon delivery that were obtained from a certified vendor (Animal Biotech Industries Inc., Danboro, PA, USA). Pigs were allowed 2 weeks of acclimation in the facility before the start of the experiment.

#### Preliminary Studies

In order to determine appropriate design of FMD transmission studies in pigs, a series of experiments were performed to establish the antemortem infection dynamics subsequent to simulated-natural inoculation of donor pigs. These experiments have been described in detail in other publications (30–32). A system of intra-oropharyngeal (IOP) deposition of 100 50% infectious doses titrated in pig heel bulbs [50% PHID; (29)] was selected based on consistent, synchronous FMD in inoculated pigs and close simulation of natural infection. A total of 15 pigs were inoculated with FMDV A24 using this dose and route combination in order to determine the duration of the incubation period (i.e., onset of clinical signs) and to estimate the inferred latent period (i.e., onset of FMDV shedding as proxy for contagiousness). The duration of these experiments ranged from 12 h to 60 days depending on study objectives. An intensive schedule of sample collection was utilized through the early phase of infection to enable detailed characterization of infection dynamics in infected pigs. In brief, samples consisting of serum and oropharyngeal (OP) swabs (see below) were collected at 4- to 6-h intervals until 24 h post-inoculation (hpi), and at 12- to 24-h intervals subsequently, with some variation in sampling time points between study cohorts.

#### Contact Transmission Trials

**Figure 1:**
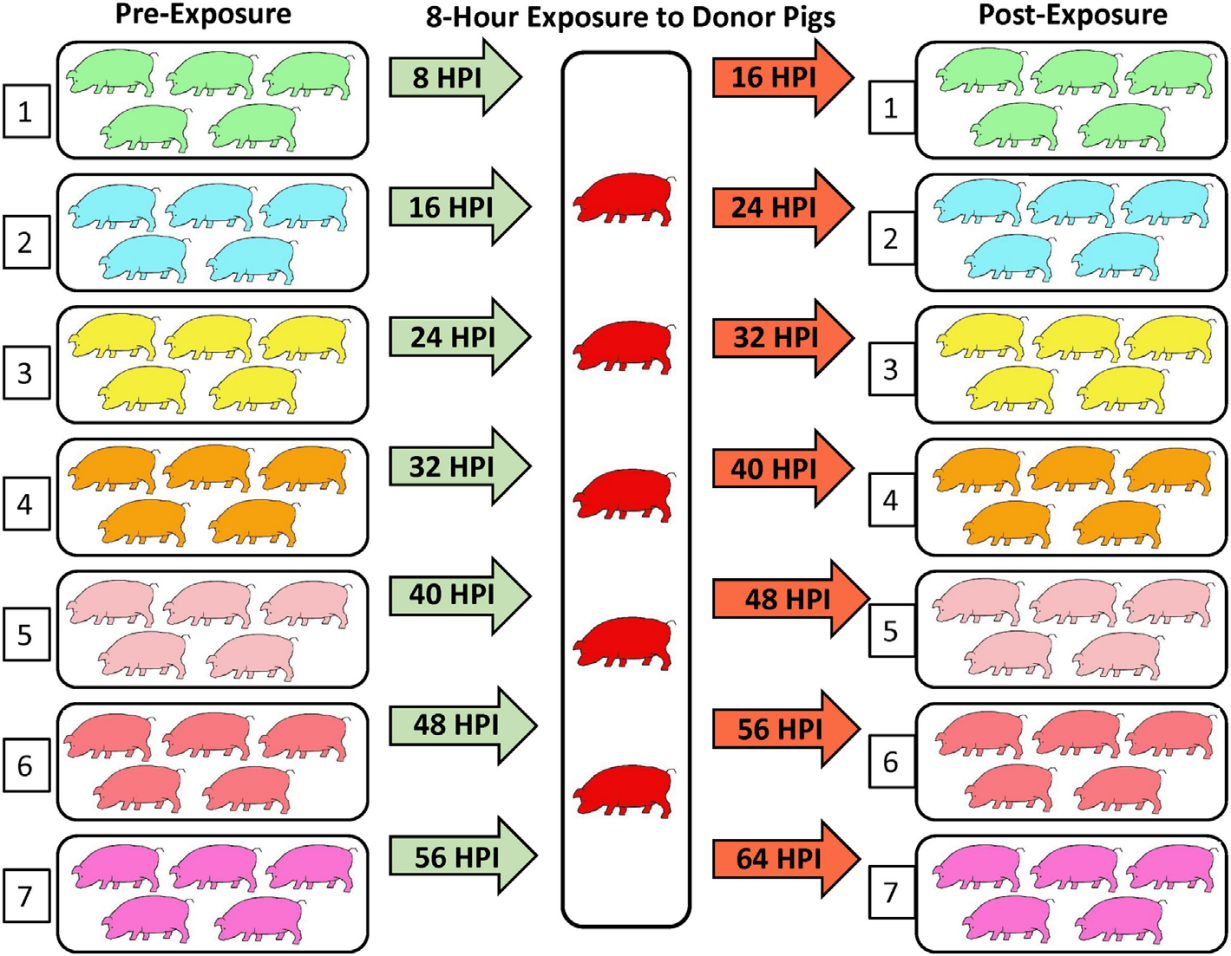
**Experimental design**. Seven groups of five pigs were comingled with five FMDV-infected donor pigs through successive 8-h time slots. The first contact group was housed together with the donor pigs from 8 to 16 h post-infection (hpi) of the donors. Contact groups were sequentially shifted in and out of the isolation room housing the donor pigs at 8-h intervals until all contact groups had been exposed to the donors at 64 hpi. Contact groups were housed in separate isolation rooms before and after exposure and were monitored for the development of FMD.

The contact transmission trial included 40 pigs that were divided into 8 groups of 5 pigs per group (Figure 1). All groups were housed in separate isolation rooms and one group (the donor pigs) was infected with FMDV A24 Cruzeiro using the optimized IOP-inoculation system (30).

Starting at 8 hpi of the donor pigs, the remaining seven groups of pigs (contact groups 1–7) were sequentially comingled with the donor pigs for periods of 8 h each (Figure 1). The donor pigs were kept in the same isolation room throughout the study, while contact groups were moved from a clean pre-exposure room into the donor room, and subsequently transferred to a different, clean post-exposure room before the subsequent contact group was moved into the donor room (Figure 1). Thus, the first contact group was exposed to the donor pigs from 8 to 16 hpi, the second group was exposed from 16 to 24 hpi, continuing similarly, until the seventh and final contact group had been exposed to the donors, at 64 hpi (Figure 1). Water was available *ad libitum* throughout the experiment. A small amount of feed was distributed on the floor of the donor’s room at the start of each contact period. Physical handling and sample collection was standardized to avoid passive transfer of virus between rooms. Animal handlers moved from clean to contaminated areas with showers and changes of clothes between rooms.

#### Clinical Evaluation and Sample Collection

Samples consisted of whole blood collected in serum separation tubes from the jugular vein and OP swabs obtained through direct swabbing of the tonsil of the soft palate using a large cotton swab. Swabs were immersed in 2-ml minimal essential media containing 25mM HEPES directly upon collection. Blood samples and OP swabs were centrifuged to extract serum and OPF, respectively. All samples were immediately frozen at −70°C until further processing.

The onset and progression of the pigs’ clinical status (lesion distribution) were quantitated using a previously described scoring system (29, 30, 33). In brief, each of 16 digits having a characteristic FMDV lesion contributed one point toward a cumulative score, with 4 additional single points added for lesions within the oral cavity, on the snout, on the lower lip, and on carpal/tarsal skin, respectively, thus resulting in an initial maximum score of 20. Individual animals’ scores were subsequently converted to a 0–5 scale to facilitate statistical comparison to other investigations performed within our laboratory. This was achieved by dividing each animal daily score by the maximum score and multiplying the fractional value by 5.

Serum and OP swabs were collected from all pigs prior to inoculation or exposure. Subsequent sampling of donor pigs consisted of collection of blood at 16, 24, 48, and 64 hpi, and OP swabs directly after inoculation and at 8-h intervals subsequently, corresponding to the time points when contact groups were moved in/out of the donor’s room. Clinical observations of donor pigs were likewise performed at 8-h intervals.

Post-exposure OP swabs were collected from contact pigs as they were transferred out of the donor’s room [8 h post-exposure (hpe)] and again at 16 and 24 hpe. Serum and OP swabs were subsequently collected at 24-h intervals (corresponding to once per day) from 24 hpe until the pigs had developed fulminant clinical FMD and were removed from the study (72–120 hpe). Pigs that did not develop any signs of FMD were sampled once daily for 10 days, with additional samples collected at 14 and 21 days post-exposure (dpe). Clinical examinations of contact pigs were performed each time samples were collected.

### FMDV RNA Detection in Serum and Swabs

**Figure 2:**
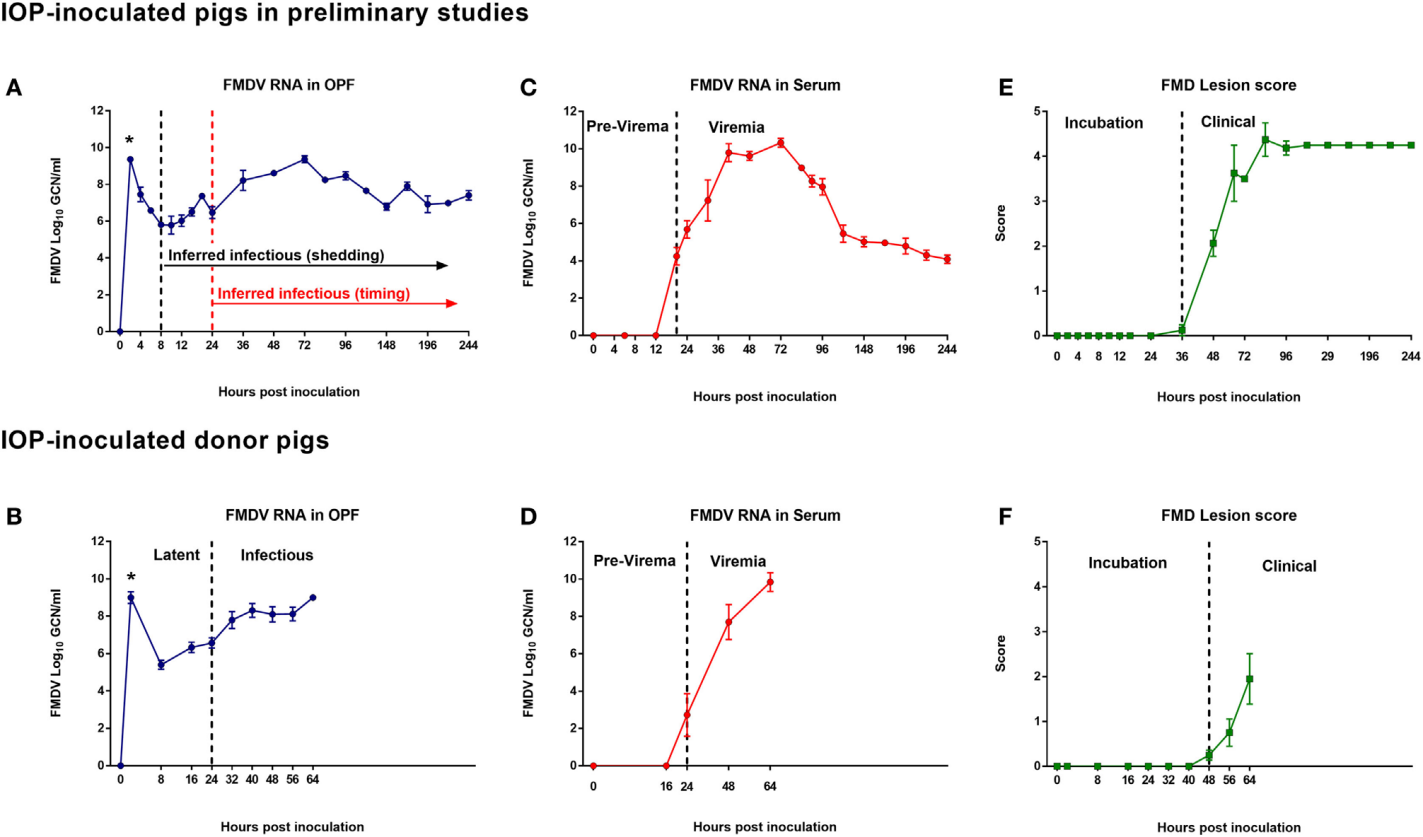
**FMDV infection dynamics in pigs inoculated *via* intra-oropharyngeal (IOP) route**. Infection dynamics in preliminary investigations (top row; *n* = 15) and subsequent transmission trial (bottom row; *n* = 5). **(A,B)** FMDV RNA in OPF determined by qRT-PCR and expressed as log_10_ genome copy numbers (GCN)/ml (mean values ± SEM). Abundant FMDV RNA measured directly after inoculation was interpreted as residual inoculum indicated by (*). Infectiousness was not determined in the preliminary experiments which had no contact exposure groups. Rather, inferred infectiousness was estimated based on onset of shedding which occurred at 8 hpi [**(A)** black dashed line; Inferred Infectiousness (shedding)]. Inferred infectiousness was also retrospectively extrapolated from the measured time of onset of infectiousness in the subsequent transmission trial [**(A)** red dashed line; Inferred Infectiousness (timing)]. The donors’ transition from latent to infectious phases of infection occurred at 24 hpi [**(B)** black dashed line] as determined by successful contact transmission of FMDV. **(C,D)** FMDV RNA in serum determined by qRT-PCR and expressed as log_10_ genome copy numbers (GCN)/ml (mean values ± SEM). Viremia was detected at 18 hpi in preliminary studies **(C)** and at 24 hpi in the donors of the transmission trial **(D)**. **(E,F)** Cumulative lesion score in preliminary investigations **(E)** and in the donors of the transmission trial **(F)**. The transition from incubation to clinical phases of infection (vertical dashed lines) occurred at 36 and 48 hpi, respectively. Assay detection limits: serum = 2.68 log_10_ GCN/ml; OPF = 3.08 log_10_ GCN/ml.

**Figure 3:**
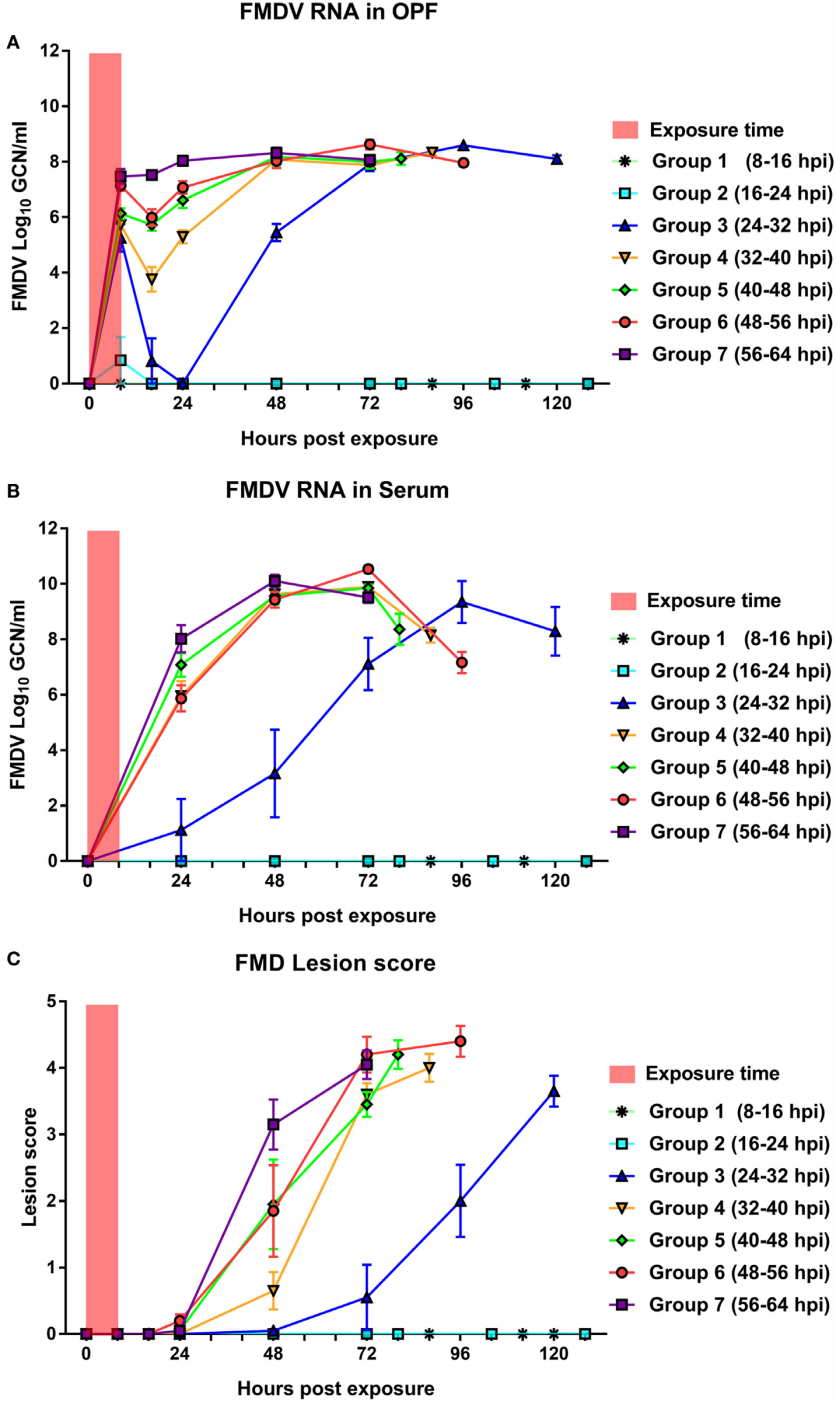
**Infection dynamics in sequentially exposed contact groups**. Quantities of FMDV RNA in OP swabs **(A)** and serum **(B)**, as well as cumulative lesion score **(C)** in seven groups of pigs that were exposed to FMDV-infected donor pigs during successive 8-h slots. Group means and SEM. There was no detection of infection in contact groups 1 and 2, which were exposed to the donors from 8 to 16 and 16 to 24 hpi, respectively. The onset and progression of infection in contact group 3 (exposed from 24 to 32 hpi) were delayed compared to subsequent groups. FMDV RNA in serum and OPF determined by qRT-PCR and expressed as log_10_ genome copy numbers (GCN)/ml (mean values ± SEM). Assay detection limits: serum = 2.68 log_10_ GCN/ml; OPF = 3.08 log_10_ GCN/ml.

Serum and OPF were analyzed using qRT-PCR, targeting the 3D region of the FMDV genome (34), as described previously (31, 32, 35, 36). Cycle threshold values were converted into FMDV genome copy numbers (GCN) per milliliter by use of a standard curve derived from analysis of 10-fold dilutions of *in vitro* synthesized FMDV RNA. The equation of the curve of GCN versus Ct values was further adjusted for dilutions used during processing of samples. Results reported in Figures 2 and 3 represent the geometric group mean (log_10_ GCN/ml ± SEM) for each time point.

### Virus Isolation

Oropharyngeal swab samples collected from contact groups 1 and 2 were cleared from debris and potential bacterial contamination by centrifugation through Spin-X^®^ filter columns (pore size 0.45 μm, Sigma-Aldrich) and were subsequently analyzed for infectious FMDV through virus isolation (VI) on LFBK αvβ6 cells (37, 38), following a protocol previously described (36). Absence of FMDV was further confirmed by qRT-PCR analysis of VI cell culture supernatants.

### Definitions

Successful transmission was determined by the detection of clinical FMD in contact-exposed pigs concurrent with viral dynamics consistent with infection. Pigs that did not develop clinical signs of FMD were kept through 21 days for the assessment of seroconversion to rule out the possibility of subclinical infection. The onset of FMDV shedding was determined as the time of the first detection of FMDV RNA in OPF that led to sustained subsequent detection. Using this definition, a single occurrence of FMDV RNA detection in OPF was not considered as virus shedding unless detection occurred in the subsequent sample. Viremia was defined by detection of FMDV RNA in serum. The onset of clinical FMD was determined as the first observed vesicular lesion in the oral cavity, on the snout or on the feet. All observations were made at individual animal level. But, transmission events could not be attributed to individual animals as contacts (*n* = 5) and donors (*n* = 5) cohabitated in the same containment unit during exposure.

### Statistical Analysis

The time to first detection of clinical FMD lesions, viremia (defined by detection of FMDV RNA in serum), and continuous shedding of FMDV RNA in OPF was compared across groups using log-rank tests with Kaplan–Meier estimated survival curves (39, 40). Computations were carried out in the statistical program R using the survival package (41). *p*-Values of ≤0.05 were considered indicative of significant differences between groups.

## Results

### Preliminary Studies

Fifteen pigs were inoculated with FMDV A24 Cruzeiro using a simulated-natural system of IOP deposition in order to estimate the parameters of incubation and latency, by determining onset of clinical disease and FMDV shedding. The selected dose and challenge route generated consistent clinical FMD and highly synchronous progression of infection in inoculated pigs (Figures 2A,C,E). Abundant but declining levels of FMDV RNA detected in OP swabs directly following inoculation were interpreted as residual inoculum. From 8 hpi, FMDV RNA detection in OPF increased, suggesting *de novo* replication of virus in the oropharynx (Figure 2A). The average detection level of FMDV RNA at the time of first detection of *de novo* shedding was 5.8 GCN/ml. Virus shedding peaked at approximately 72 hpi before gradually declining. Viremia, defined by detection of FMDV RNA in serum, was first detected at 18 hpi and peaked at 72 hpi (Figure 2C). A single vesicular lesion was detected at 36 hpi in one animal, with lesions appearing between 48 and 72 hpi in the remaining pigs (Figure 2E). Based on these data, it was determined that under these conditions, the transition from preshedding to shedding (inferred infectious) phases of infection occurred at 8 hpi (Figure 2A), while the transition from incubation to clinical phase occurred at 36–48 hpi (Figure 2E). On the basis of these preliminary experiments, it was determined that, in order to dissect incubation and latent periods under the current experimental conditions, a transmission experiment would have to span from a minimum of 8–48 hpi.

### Contact Transmission Trial

#### Infection Dynamics in IOP-Inoculated Donor Pigs

Five pigs designated as virus donors were inoculated with FMDV A24 Cruzeiro by the IOP route, as described previously (30, 31). Overall, infection dynamics were highly similar to the preliminary experiments (Figure 2). Adequate deposition of inoculum was confirmed by detection of abundant quantities of virus measured in OP swabs directly following inoculation (“*” in Figure 2B). After clearance of residual inoculum, FMDV shedding was detected in OPF of all five donor pigs at 8 hpi, with a mean value of 5.22 log_10_ GCN/ml. FMDV shedding in OPF increased continuously reaching a maximum level of 9.0 log_10_ GCN/ml at 64 hpi at which time the donor pigs were euthanized (Figure 2B). FMDV RNA was detected in serum at 24 hpi in three out of five donor pigs, and at 48 hpi in the remaining two donor pigs. The maximum mean serum concentration of 9.85 log_10_ GCN/ml was measured at the time of euthanasia (64 hpi; Figure 2D). Early clinical signs of FMDV, including subtle blanching and vesiculation at coronary bands and tongue, were observed in three donor pigs at 48 hpi, and one pig at 56 hpi; cumulative lesion scores in these four pigs progressed gradually until the final assessment at 64 hpi (Figure 2F). One pig did not develop FMD lesions within the study period; however, successful infection of this pig was confirmed by detection FMDV RNA in OPF and blood at 8 and 48 hpi, respectively.

#### Contact Groups 1 and 2

**Figure 4:**
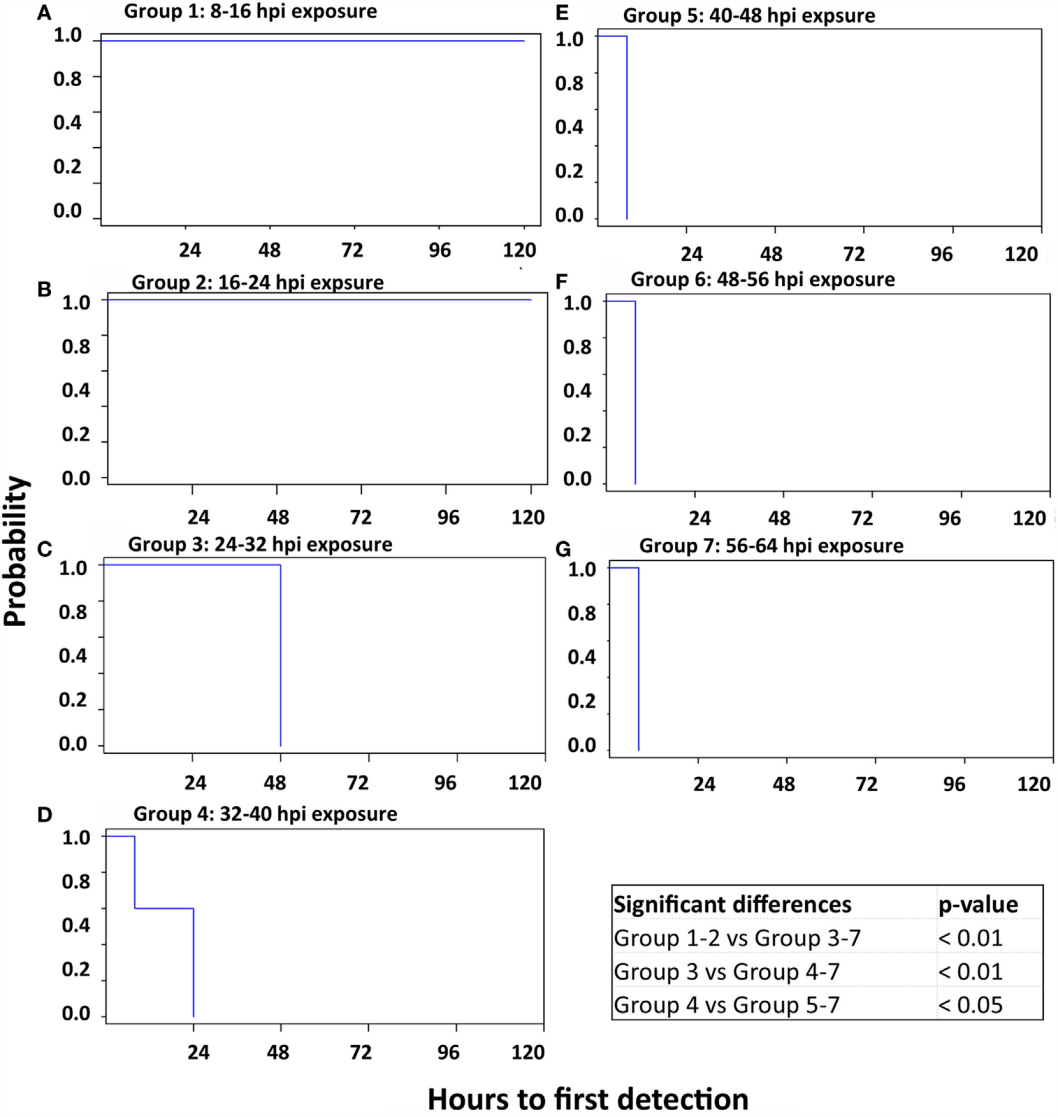
**Kaplan–Meier survival curves; time to detection of OP shedding of FMDV**. Survival curves delineating the time to onset of oropharyngeal shedding of FMDV RNA in seven sequentially exposed groups of pigs. There was no shedding of FMDV in groups 1 and 2 (A and B), which were thus significantly different from all other groups. Additionally, the onset of FMDV shedding was significantly delayed in groups 3 and 4 compared to all subsequent groups. **(A)** Group 1: 8–16 hpi exposure. **(B)** Group 2: 16–24 hpi exposure. **(C)** Group 3: 24–32 hpi exposure. **(D)** Group 4: 32–40 hpi exposure. **(E)** Group 5: 40–48 hpi exposure. **(F)** Group 6: 48–56 hpi exposure. **(G)** Group 7: 56–64 hpi exposure.

**Figure 5:**
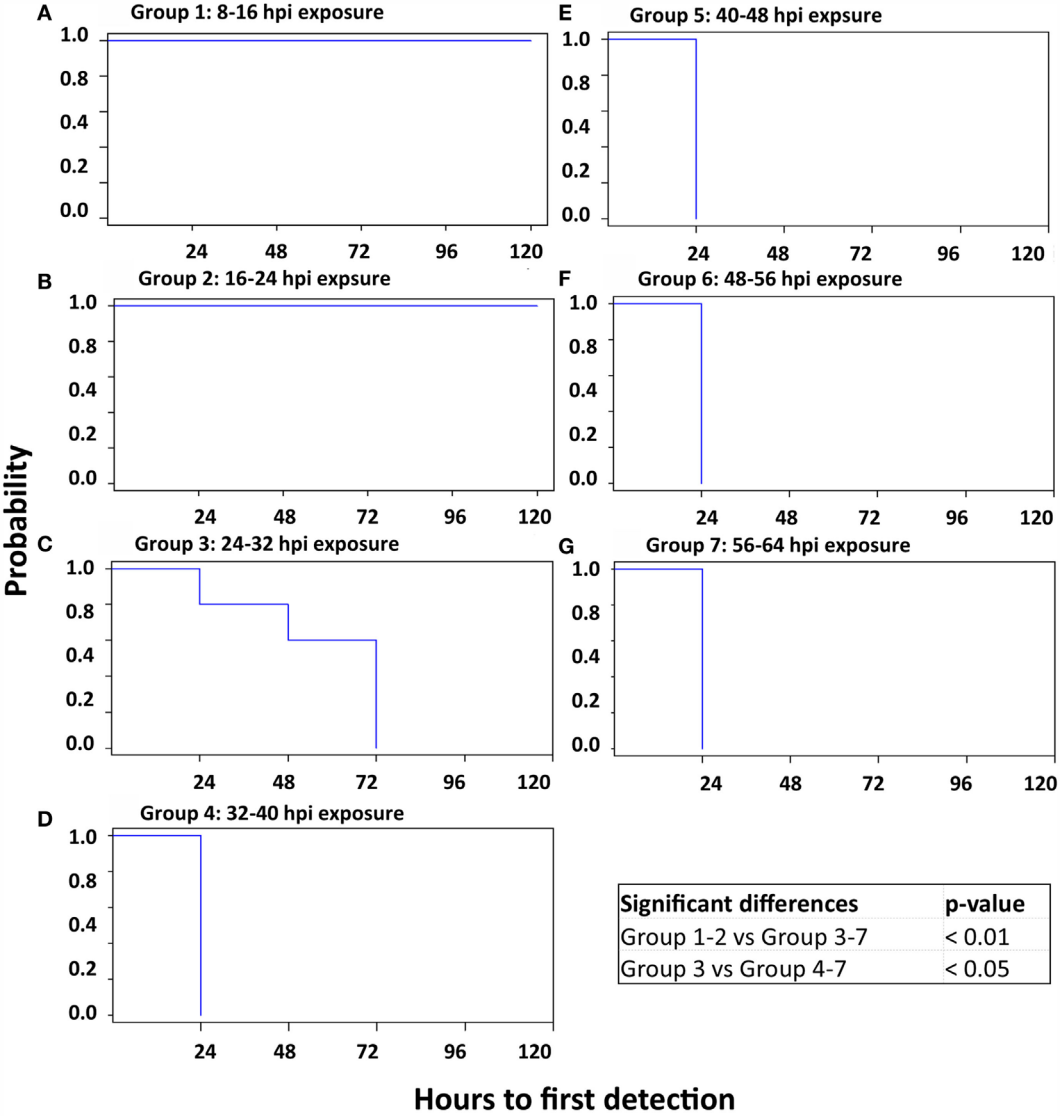
**Kaplan–Meier survival curves; time to detection of viremia**. Survival curves delineating the time to onset of viremia, defined by detection of FMDV RNA in serum. Seven groups of pigs were sequentially exposed to FMDV-infected donor pigs. There was no detection of FMDV in serum in groups 1 and 2 **(A,B)**, which were thus significantly different from all other groups. Additionally, the onset of FMDV viremia was significantly delayed in group 3 compared to subsequent groups. **(A)** Group 1: 8–16 hpi exposure. **(B)** Group 2: 16–24 hpi exposure. **(C)** Group 3: 24–32 hpi exposure. **(D)** Group 4: 32–40 hpi exposure. **(E)** Group 5: 40–48 hpi exposure. **(F)** Group 6: 48–56 hpi exposure. **(G)** Group 7: 56–64 hpi exposure.

**Figure 6:**
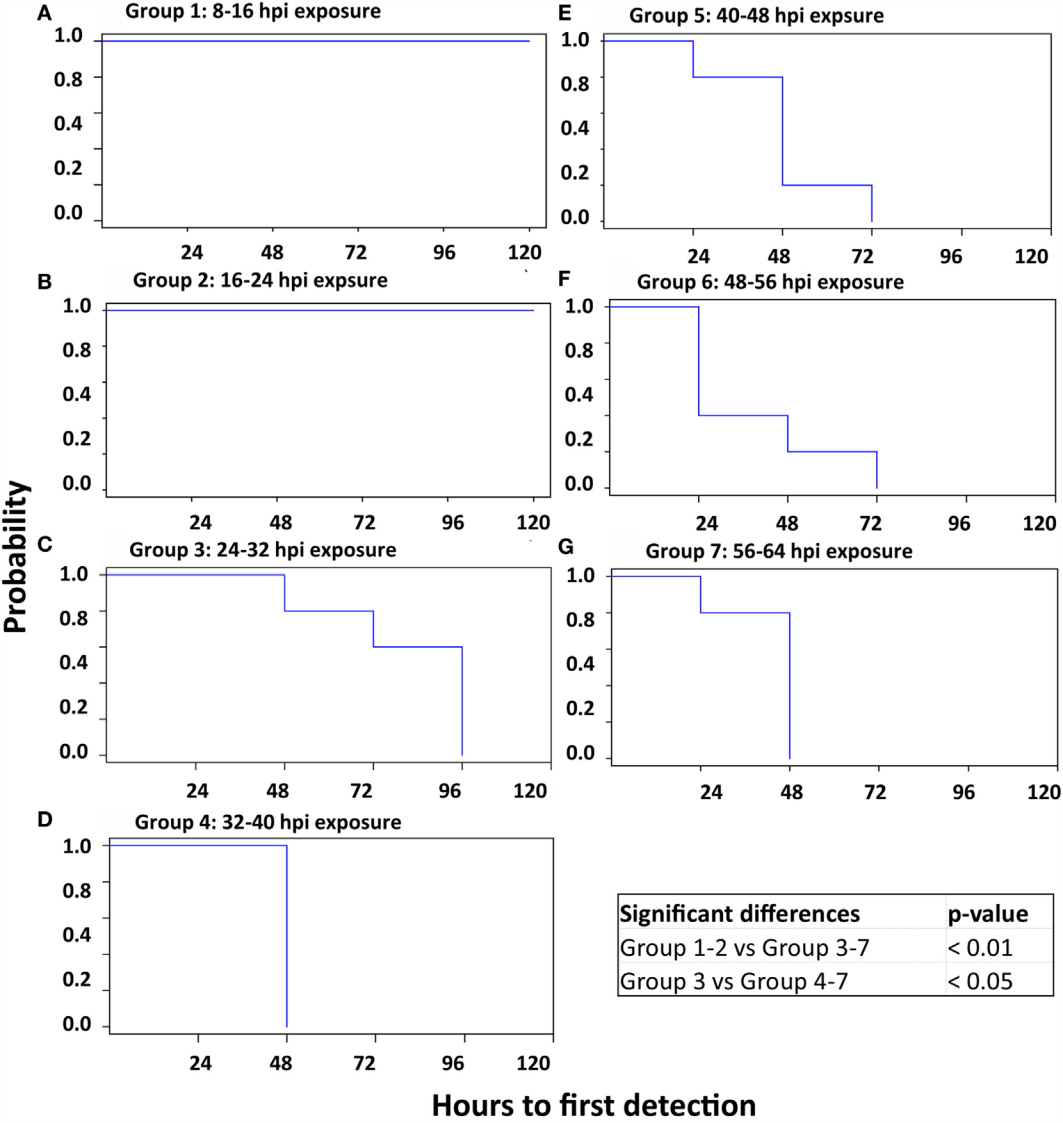
**Kaplan–Meier survival curves; time to detection of FMD lesions**. Survival curves delineating the time to detection of vesicular FMD lesions in seven sequentially exposed groups of pigs. There were no FMD lesions in groups 1 and 2 **(A,B)**, which were thus significantly different from all other groups. Additionally, the onset of clinical FMD was significantly delayed in group 3 compared to subsequent groups. **(A)** Group 1: 8–16 hpi exposure. **(B)** Group 2: 16–24 hpi exposure. **(C)** Group 3: 24–32 hpi exposure. **(D)** Group 4: 32–40 hpi exposure. **(E)** Group 5: 40–48 hpi exposure. **(F)** Group 6: 48–56 hpi exposure. **(G)** Group 7: 56–64 hpi exposure.

Contact group 1 cohabitated with the donor pigs from 8 to 16 hpi, whereas contact group 2 was subsequently exposed to the donors from 16 to 24 hpi (Figure 1). Detection of FMDV RNA in OPF of donor pigs was 5.22 log_10_ GCN/ml at the start of contact group 1 exposure, and 6.51 log_10_ GCN/ml at the end of contact group 2 exposure (Table 1). None of the 10 pigs in contact groups 1 or 2 developed any signs of FMDV infection (Figures 3–6; Table 1). Low quantities of FMDV RNA were detected in OP swabs of one pig in contact group 2 at 8 hpe, corresponding to the end of the contact exposure. FMDV RNA was not detected in any subsequent OPF or serum samples collected from the pigs in either of these two groups (Figure 3). Pigs in groups 1 and 2 were monitored through 21 dpe. Serum samples collected at 21 dpe did not contain neutralizing antibodies against FMDV, confirming that the pigs had not been subclinically infected (not shown).

**Table 1:** **Progression of infectiousness in pigs inoculated with FMDV *via* intra-oropharyngeal (IOP) route**.

Contact group	Exposure time^a^ (hpi)	Donor pig characteristics during exposure				Infected contact pigs (infected/not infected)	Infectious phase of donors
		Viremia	Clinical FMD	FMDV RNA in OPF^b^			
				Start exposure	End exposure		
1	8–16	No	No	5.22	6.45	0/5	Latent
2	16–24	No	No	6.45	6.51	0/5	Latent
3	24–32	Yes	No	6.51	7.22	5/0	Infectious *(incubation)*
4	32–40	Yes	No	7.22	8.31	5/0	Infectious *(incubation)*
5	40–48	Yes	Yes	8.31	8.10	5/0	Infectious *(clinical)*
6	48–52	Yes	Yes	8.10	8.10	5/0	Infectious *(clinical)*
7	52–64	Yes	Yes	8.10	9.00	5/0	Infectious *(clinical)*

*^a^Expressed as hours post-infection of donors*.

*^b^Expressed as log_10_ GCN/ml*.

#### Contact Group 3

The five pigs in contact group 3 were exposed to the donor pigs from 24 to 32 hpi. During this period, donor pigs were viremic, but had not yet developed fever (not shown) or vesicular FMD lesions (Figure 2). FMDV RNA detection in OPF of the donors was 6.51 log_10_ GCN/ml at the start of exposure, and 7.22 log_10_ GCN/ml when contact group 3 was removed from the room (Table 1). FMDV RNA was detected in OPF of all five pigs in groups 3 at the end of the exposure period (8 hpe; Figure 3A), with a mean value of 5.25 log_10_ GCN/ml. However, FMDV RNA was only detected in OPF from one out of the five pigs at the subsequent time point (16 hpe), and OPF from all five pigs were below the limit of detection for FMDV RNA at 24 hpe (Figure 3A). Viral shedding in OPF was again detected in all pigs at 48 hpe and gradually increased to a peak mean value of 8.59 log_10_ GCN/ml measured at 96 hpe (Figure 3A). FMDV RNA in serum was detected at 24 hpe in one pig, at 48 hpe in the second pig, and at 72 hpe in the remaining three pigs (Figure 3B). Vesicular lesions were detected in all five pigs between 48 and 96 hpe (24 h after the first detection of FMDV RNA in serum). There was a continuous increase in cumulative lesion scores until the pigs were euthanized at 120 hpe (Figure 3C).

#### Contact Group 4

The five pigs of contact group 4 cohabitated with the donor pigs from 32 to 40 hpi. Similar to the previous exposure period, the donor pigs were viremic during the contact period, but without any clinical signs of FMD (Figure 2; Table 1). Mean detection of FMDV RNA in OPF of the donors was 7.22 log_10_ GCN/ml at the initiation of exposure, and 8.31 log_10_ GCN/ml at the end of exposure (Table 1; Figure 2D). FMDV RNA was detected in OPF from all five pigs in contact group 4 at the end of the exposure period (8 hpe), with a mean value of 5.72 log_10_ GCN/ml (Figure 3A). Virus detection in OPF was negative in three out of the five pigs at 16 hpe, while shedding was continuous in two pigs. There was subsequently a consistent increase in FMDV RNA levels in OPF, from 24 hpe until a maximum mean value of 8.33 log_10_ GCN/ml was measured before the pigs were euthanized at 88 hpe (Figure 3A). FMDV RNA was detected in serum of all five pigs at 24 hpe, with serum levels reaching a maximum mean value of 9.89 log_10_ GCN/ml at 72 hpe (Figure 3B). Vesicular lesions were detected at 48 hpe in all five pigs, with cumulative lesions scores consistently increasing until the time of euthanasia (Figure 3C).

#### Contact Group 5

The five pigs in contact group five were exposed to the donor pigs from 40 to 48 hpi which corresponded to the end of the incubation period and transition to the clinical phase of infection for the donor group. There were no clinical signs of FMD in the donor pigs at the start of the contact group 5 exposure slot (40 hpi), but three out of the five donor pigs had developed vesicular lesions by 48 hpi (Figure 2F). Mean shedding of FMDV RNA in OPF of the donors was 8.31 log_10_ GCN/ml at the start of group 5 exposure, with a marginal decrease to 8.10 log_10_ GCN/ml at the end of exposure (Table 1; Figure 2D). Mean FMDV RNA detection in OPF from contact group 5 at the end of the exposure (8 hpe) was 6.13 log_10_ GCN/ml (Figure 3A). This quantity had decreased marginally to 5.72 log_10_ GCN/ml at 16 hpe, but shedding was continuous in all five pigs. Peak shedding (8.18 log_10_ GCN/ml) was detected at 48 hpe, and virus shedding was sustained close to that level until the pigs were euthanized at 80 hpe (Figure 3A). All five pigs were viremic at 24 hpe, with peak serum concentration of virus (9.84 log_10_ GCN/ml) measured at 72 hpe (Figure 3B). One pig had a clearly demarcated coronary band vesicle at 24 hpe, and clinical lesions were detected at 48 hpe in the other four pigs. Cumulative lesion scores progressed rapidly, and all pigs had severe clinical FMD and were unwilling to stand and/or move at the time of euthanasia (80 hpe) (Figure 3C).

#### Contact Group 6

Contact group 6 was exposed to the donors from 48 to 56 hpe. Four out of the five donor pigs had early signs of clinical FMD during this time frame. The mean quantities of FMDV RNA detected in OPF of the donors were 8.10 log_10_ GCN/ml at the beginning and at the end of group 6 exposure. Mean OPF detection in the contact pigs at the end of the exposure period (8 hpe) was 7.15 log_10_ GCN/ml. Similar to contact group 5, there was a modest drop in OPF detection in contact group 6 pigs by 16 hpe (5.99 log_10_ GCN/ml). Shedding was continuous in all five pigs, with a peak mean value of 8.63 log_10_ GCN/ml at 72 hpe (Figure 3A). All pigs were viremic at 24 hpe, with peak mean serum concentration of virus (10.53 log_10_ GCN/ml) measured at 72 hpe (Figure 3B). Clinical lesions appeared at 24 hpe (three pigs), 48 hpe (one pig), or 72 hpe (one pig) (Figure 3C). Similar to the preceding contact group, all pigs were severely affected by the clinical disease starting at 48–72 hpe and were euthanized at 96 hpe.

#### Contact Group 7

Contact group 7 was exposed to the donors from 56 to 64 hpi. The clinical status of the donor group was similar to the previous exposure period, with vesicular lesions in four out of the five pigs (Figure 2F). Mean shedding in OPF of donor pigs was 8.10 log_10_ GCN/ml at the beginning of the exposure and 9.00 log_10_ GCN/ml at the end of the contact period (Table 1; Figure 2B). The mean OPF detection of FMDV RNA in contact pigs at the end of exposure was 7.47 log_10_ GCN/ml, and OPF shedding increased continuously until maximum values of 8.32 log_10_ GCN/ml were measured at 48 hpe (Figure 3A). The mean serum concentration of FMDV RNA at 24 hpe was 8.02 log_10_ GCN/ml, with an increase to a peak average value of 10.10 at 48 hpe (Figure 3B). Clinical lesions were detected at 24 hpe in one pig and 48 hpe in four pigs (Figure 3C). The pigs in group 7 were euthanized at 72 hpe due to the severity of clinical FMD.

### Statistical Comparison of Time to Onset of Viremia, Shedding, and Clinical Disease across Contact Groups

The time to event for onset of important disease dynamic parameters were compared across contact groups in order to characterize differences associated with the conditions of contact exposure. An omnibus test indicated that there were significant differences among the Kaplan–Meier estimated survival curves for the seven contact groups at the 0.05 significance level in the elapsed times from contact exposure until the first detection of FMDV shedding in OPF, onset of viremia, and appearance of clinical FMD lesions across contact groups. Specifically, the onset and progression of these indicators of infection were more rapid in contact groups that had been exposed to the donors during later stages of infection. Contact groups 1 and 2, which did not become infected with FMDV through contact were, as expected, significantly different pairwise from all other groups in all three parameters evaluated (Figures 4–6). Contact group 3, which had been exposed to the donors from 24 to 32 hpi, was also significantly different compared to groups 4–7, pairwise, for all parameters as FMDV shedding in OPF (Figure 4), viremia (Figure 5), and clinical lesions (Figure 6) were delayed relative to subsequent contact groups. Additionally, detection of FMDV shedding in OPF occurred significantly later in contact group 4 (32–40 hpi exposure) relative to later groups (Figure 4), whereas this group was not different from later groups with regard to detection of viremia or clinical lesions (Figures 5 and 6).

## Discussion

The highly contagious nature of FMDV can be attributed to a combination of factors including broad host range, low infectious dose, and shedding of large quantities of virus by infected animals (3, 4, 42, 43). Even though FMDV transmission may occur *via* both direct (animal to animal) and indirect (mechanical transfer and airborne spread) mechanisms, the most significant risk for dissemination of disease during the early phase of an outbreak is transport of infected animals (44). The extent of spread during the early, high risk period of an FMD outbreak can be influenced by the occurrence of mild or unrecognized clinical symptoms of disease, as well as the potential for disease transmission from infected animals that have not yet developed clinical disease (13). The current investigation was designed for the purpose of determining the onset of infectiousness (end of latent period) in relation to the appearance of clinical signs (end of incubation period) in pigs infected with FMDV. Different characteristics of infection dynamics were considered for appropriateness as proxies for contagiousness for modeling purposes. An intensive schedule of sampling and clinical observations in combination with continuous, sequential exposure of contact pigs to donors enabled detailed characterization of the progressive transmission dynamics of FMDV in groups of pigs.

The experimental design was based on previous experience of characterizing infection dynamics of this specific FMDV strain in pigs (23, 30–32). The donor pigs were infected using a simulated-natural system of IOP inoculation (30, 31) and optimal timing of contact exposure was determined based on data obtained from a series of preliminary experiments utilizing the same virus and challenge system. The IOP-inoculation system has the advantage of utilizing a natural route of FMDV exposure for pigs while also facilitating precise control of the timing and dose of virus challenge. Additionally, as this is a needle-free inoculation system, there are no primary vesicles at injection sites that could constitute an additional (artificial) source of virus exposure for contact animals.

Foot-and-mouth disease virus shedding was continuously detected in OPF from all pigs in the donor group from the time of inoculation until termination of their monitoring period. Despite this continuous detection, there was no transmission of FMDV to any of the pigs in the first two contact groups, which were exposed to the donors from 8 to 16 and 16 to 24 hpi, respectively. On this basis, the transition from latency to infectiousness within the donor group was determined to have occurred between 24 and 32 hpi as all contact pigs that were exposed to the donors from this time and onward developed severe clinical FMD within 1–3 days after exposure. There were, however, no clinical signs of FMD detected in any of the donor pigs before 48 hpi. Thus, pigs in contact groups 3 and 4 (exposed from 24 to 32 and 32 to 40 hpi, respectively) were infected, while all of the donor pigs were still within the incubation (preclinical) phase of infection. The difference between detection of shedding versus confirmed infectiousness indicated that presence of FMDV in donor pigs was not sufficient to define infectiousness and suggests that a threshold quantity of shedding is required for FMDV to be transmitted.

Successful transmission of FMDV occurred concurrently with the first detection of viremia in the donor pigs. Even though the presence of virus in the blood can be assumed to not have any causal relationship to transmission of infection, the current and previous investigations have demonstrated that occurrence of viremia is associated with a concurrent surge in virus shedding *via* the OP route (23, 31, 45). These data suggest that onset of viremia and threshold-defined shedding of FMDV are better proxies for infectiousness than onset of clinical disease (end of incubation period).

There was moderate variation in the onset of viremia and the first detection of clinical lesions among pigs within the earliest contact groups to get infected (group 3), whereas the variation in infection dynamics was lower within later groups (groups 4 through 7). FMDV shedding in group 3 was low but largely consistent across animals through the early time points after exposure. Specifically, FMDV shedding in OPF was below the limit of detection at 24 hpe in all five pigs of contact group 3. This modest and relatively synchronous FMDV shedding in OPF through 8–48 hpe suggests that all five pigs in group 3 did likely get infected directly by the donors. However, due to limitations of the study design, it was not possible to rule out the possibility of within-group transmission in this group. Contrastingly, the highly synchronous infection dynamics within subsequent contact groups strongly suggested direct transmission from donors to contact pigs.

All pigs in contact groups 3 through 7, which were exposed to donors from 24 hpi and later, developed similarly severe FMD characterized by high-titer viremia and vesicular lesions on all four feet as well as in the oral cavity or on the snout. Thus, there was no difference in disease severity between the pigs in any of these groups as they reached the pre-determined end point of the study. There were, however, more pronounced differences between these (infected) contact groups at earlier time points after exposure as was reflected in the significant differences in the time to event analyses. While the later contact groups (groups 5 through 7) had a very rapid onset of severe FMD, the progression of clinical disease was slower and more gradual in contact group 3. This finding is consistent with previous investigations which have described similar associations between increased challenge dose and a shorter time to onset of viremia and clinical lesions in FMDV-exposed pigs (25, 28, 30). In the current study, the effective challenge dose of successive contact groups was reflected by the quantity of FMDV detected in OPF from the donor pigs, which steadily increased from 8 to 40 hpi. The increase in FMDV shedding by donors through subsequent exposure periods (40–64 hpi) was less pronounced. However, the appearance of vesicular lesions, containing high loads of amplifying virus, by 48 hpi would have contributed to a progressively higher challenge dose for the later challenge groups. It is noteworthy that the progression of viremia and clinical FMD in contact groups 5 through 7 was faster than in the directly inoculated donors, suggesting that the contact challenge received by these groups was greater than the IOP-delivered dose. This is consistent with the concept that direct contact exposure, albeit of limited duration, is a highly stringent challenge system for FMDV studies in pigs [reviewed in Ref. (46)].

A previous study by Quan et al. (26) concluded that although there was a progressive increase in infectiousness and FMDV transmission over time when pigs were exposed in groups, this was not the case when contact pigs were individually exposed to infected donors. Specifically, there was very limited transmission of FMDV when one contact pig was exposed to one donor pig, regardless of the infectious state of the donor pig. Similarly, a previous investigation from our laboratory demonstrated that the duration of contact exposure had substantial influence on FMDV transmission within groups of pigs, and that the effect of altered exposure duration was strain specific (23). The combined conclusions of these previous works accentuate the critical influence of experimental design on the outcome and interpretation of transmission experiments. The lack of evaluation of individual (one-on-one) exposure of contact pigs in the current study limits the ability to attribute FMDV transmission to specific individuals or to precisely measurable shedding parameters. However, pigs are generally not housed individually, or in pairs, under commercial production conditions; therefore, estimation of transmission proxies based on isolated individuals could underestimate parameters for modeling of disease spread in natural settings.

The results of this study suggest that FMDV shedding parameters may be better proxies for FMDV transmission than clinical signs of disease under these specific experimental conditions. This finding differs from the conclusions published by Charleston et al. in 2011, which were based on an experimental design investigating transmission of serotype O FMDV between calves that were housed in pairs (one-on-one exposure) (16). Furthermore, our findings suggest that FMDV transmission occurred when the mean levels of FMDV shedding in OPF within the donor group exceeded a distinct threshold (6.50 log_10_ GCN/ml ± 0.58). Thus, OP shedding of FMDV in pigs should not be treated as a categorical variable indicative of infectiousness. This is specifically relevant to meta-analyses conducted to obtain infection parameters (i.e., estimation of latent and infectious periods) that feed mathematical modeling, which have not traditionally incorporated this concept.

The current investigation demonstrated transmission of FMDV during the incubation period of pigs housed in groups. The transition from latent to infectious phases of disease occurred approximately 24 h prior to the appearance of clinical signs of disease. There was a progressive increase in infectiousness of donor pigs through the acute phase of disease as the onset and progression of clinical FMD in contact pigs were faster in groups that were exposed to the donors during advanced stages of clinical FMD, which is consistent with an increased effective challenge dose. These findings should be considered for modeling of FMDV outbreaks involving pigs. Similar studies performed in other susceptible species may provide additional insights to the relationships between incubation and latency of FMDV infection.

## Author Contributions

CS contributed to study design, coordinated and executed the animal experiments, and drafted the manuscript. JP contributed to study design and execution of the animal experiments, and oversaw laboratory analyses. BB performed statistical analyses and interpretation of data. K-MT performed statistical analyses and interpretation of data. MB performed statistical analyses and interpretation of data. AD coordinated and oversaw data analyses and contributed scientific content. LR contributed to study design and scientific content. JA conceived and coordinated the work, contributed to writing the manuscript, and promulgated addition of vertical lines in Figure 2. All the authors have critically reviewed and revised the manuscript and approved the final product.

## Conflict of Interest Statement

The authors declare that the research was conducted in the absence of any commercial or financial relationships that could be construed as a potential conflict of interest. The reviewer LL and handling Editor declared their shared affiliation, and the handling Editor states that the process nevertheless met the standards of a fair and objective review.
